# Splitting the P-Wave: Improved Evaluation of Left Atrial Substrate Modification after Pulmonary Vein Isolation of Paroxysmal Atrial Fibrillation

**DOI:** 10.3390/s22010290

**Published:** 2021-12-31

**Authors:** Aikaterini Vraka, Vicente Bertomeu-González, Fernando Hornero, Aurelio Quesada, Raúl Alcaraz, José J. Rieta

**Affiliations:** 1 BioMIT.org, Electronic Engineering Department, Universitat Politecnica de Valencia, 46022 Valencia, Spain; aivra@upv.es; 2 Clinical Medicine Department, Miguel Hernández University, 03202 Elche, Spain; vbertomeu@umh.es; 3 Cardiovascular Surgery Department, Hospital Clínico Universitario de Valencia, 46010 Valencia, Spain; hornero_fer@gva.es; 4 Arrhythmia Unit, Cardiology Department, General University Hospital Consortium of Valencia, 46014 Valencia, Spain; quesada_aur@gva.es; 5 Research Group in Electronic, Biomedical and Telecommunication Engineering, University of Castilla-La Mancha, 16071 Cuenca, Spain; raul.alcaraz@uclm.es

**Keywords:** atrial fibrillation, pulmonary vein isolation, atrial substrate modification, P-wave, left atrium

## Abstract

Atrial substrate modification after pulmonary vein isolation (PVI) of paroxysmal atrial fibrillation (pAF) can be assessed non-invasively by analyzing P-wave duration in the electrocardiogram (ECG). However, whether right (RA) and left atrium (LA) contribute equally to this phenomenon remains unknown. The present study splits fundamental P-wave features to investigate the different RA and LA contributions to P-wave duration. Recordings of 29 pAF patients undergoing first-ever PVI were acquired before and after PVI. P-wave features were calculated: P-wave duration (PWD), duration of the first ($PWD_{on\text{-}peak}$) and second ($PWD_{peak\text{-}off}$) P-wave halves, estimating RA and LA conduction, respectively. P-wave onset ($PW_{on}\text{-}R$) or offset ($PW_{off}\text{-}R$) to R-peak interval, measuring combined atrial/atrioventricular and single atrioventricular conduction, respectively. Heart-rate fluctuation was corrected by scaling. Pre- and post-PVI results were compared with Mann–Whitney U-test. PWD was correlated with the remaining features. Only PWD (non-scaling: Δ=−9.84%, p=0.0085, scaling: Δ=−17.96%, p=0.0442) and $PWD_{peak\text{-}off}$ (non-scaling: Δ=−22.03%, p=0.0250, scaling: Δ=−27.77%, p=0.0268) were decreased. Correlation of all features with PWD was significant before/after PVI (p<0.0001), showing the highest value between PWD and $PW_{on}\text{-}R$ ($\rho_{max}=0.855$). PWD correlated more with $PWD_{on\text{-}peak}$ (ρ= 0.540–0.805) than $PWD_{peak\text{-}off}$ (ρ= 0.419–0.710). PWD shortening after PVI of pAF stems mainly from the second half of the P-wave. Therefore, noninvasive estimation of LA conduction time is critical for the study of atrial substrate modification after PVI and should be addressed by splitting the P-wave in order to achieve improved estimations.

## 1. Introduction

With a fast-growing incidence and prevalence around the world, atrial fibrillation (AF) is currently the most common cardiac arrhythmia [1]. It is additionally connected with a plenty of other comorbidities, which can augment the hospitalization duration and frequency and affect significantly the patients’ quality of life [1]. AF is considered a supraventricular tachyarrhythmia, with desynchronized atrial electrical activations triggered principally in the pulmonary veins (PVs) and being propagated all over the atria. As a consequence, AF is characterized in the electrocardiogram (ECG) by the absence of discrete P-waves which are replaced by low-amplitude and irregular fibrillatory waves, causing a faster and irregular heart rate [1,2].

As AF initiation is mainly attributed to the pulmonary veins (PVs), PV isolation (PVI) is considered the star AF treatment [1,3]. Paroxysmal AF patients, suffering from short AF episodes that cardiovert spontaneously in less than 7 days, benefit especially from PVI, probably because arrhythmogenic activity is often limited to PVs [1,4]. Nevertheless, persistent AF patients, showing longer AF episodes that may significantly affect the atrial structure and function, may not respond positively to the PVI procedure in the long term, requiring repeated PVI sessions in some cases [1,4,5]. Apart from PVs, various right (RA) and left atrial (LA) sites additionally contribute to the AF perpetuation due to fibrosis, forming the so-called atrial substrate [6,7,8,9,10,11]. Frequent non-PV triggers are crista terminalis, interatrial septum, LA posterior wall, LA appendage, coronary sinus and ligament of Marshall. These triggers can be detected by studying the activation patterns with the help of multipolar mapping and recording catheters placed in coronary sinus and PVs [6]. When non-PV triggers are detected, focal ablation of the sites provoking the AF activation in combination with PVI is suggested in order to obtain improved results [6].

While PVI and focal ablation can eliminate AF triggers, AF propagation can be further sustained due to changes in the atrial substrate, known as atrial remodeling [1,12]. Remodeling can take place at the structural, functional or electrical level, either showing different mechanisms that contribute to the AF perpetuation. Atrial fibrosis is the common denominator of these changes [12,13]. In this respect, additional ablation based on specific electrogram characteristics that possibly indicate the fibrotic tissue is performed sometimes [14] with the assistance of automatic AF fractionation estimators [15] or by the localization of low-voltage areas, which indicate the presence of atrial scar [16,17]. Notwithstanding, extensive ablation can have adverse effects in individuals and should be sparse and in any case performed with caution [18]. Hence, meticulous atrial mapping is a necessary step during each ablation procedure whether or not non-PV ablation takes place. With that being said, the AF confrontation is not limited to PVI but requires a detailed and in depth analysis of the substrate modification provoked by PVI, in order to plan efficiently the personalized follow-up strategy and reduce the possibility of AF recurrence [19].

PVI procedure is performed by electrically isolating the PVs, in order to impede the transmission of ectopic and chaotic electrical activities towards the atria. The effect of PVI in the atrial anatomy is mainly observed in the LA, where recovery of normative atrial function, known as reverse remodeling, takes place, indicating a favorable PVI outcome [1,3,12,20,21]. Timely detection of any changes implying the presence or absence of reverse remodeling is critical in decision-making regarding the next step after PVI, as additional ablation or other strategies should be adopted in order to avoid early AF recurrence, an ominous marker of a non-successful procedure [1].

Changes in the atrial substrate can be assessed noninvasively by the analysis of the P-waves, which are the atrial component of the surface ECG. In fact, a vast amount of studies focus on analyzing the P-wave duration, which describes the atrial conduction time throughout the atria [20]. Long or very short P-waves and long P-R interval are connected with conduction slowing due to fibrosis or shortening of atrial refractoriness and are predictors of AF recurrence [12,22,23,24,25,26,27,28,29,30]. Therefore, the hypothesis that P-wave shortening indicates a less heterogeneous tissue and a favorable PVI outcome has been supported by many studies [20,26,27,31,32]. P-wave duration is also useful in predicting the AF onset, with higher variability and longer P-waves alerting the possibility of a forthcoming AF episode [33]. A significant advantage of the P-waves analysis is that it allows atrial substrate modification assessment as early as the moment after the end of the PVI session, in contrast with other techniques that require some blanking period [21].

Despite the usefulness of P-wave analysis in atrial substrate alteration assessment, there is still room for improvement. Difference in thresholds defining a prolonged P-wave can induce confusion and significantly affect the decision for the follow-up treatment to be adopted [22,23,29,34]. While agreement of a universal threshold would be the optimal solution, differences in study population as well as P-wave delineation sensitivity complicate significantly this task. With the P-wave analysis gaining more and more popularity, attention to detail may enhance the noninvasive evaluation of substrate modification after PVI. P-wave consists of two parts. The first part, from the onset to the peak of the P-wave, corresponds to the depolarization of the RA. Activation is propagated from the RA to the LA through Bachmann bundle or the area proximal to coronary sinus, thus yielding the second part of the P-wave [35,36].

Although remodeling can be present in both atria [10], it is LA which is principally affected from substrate modification after PVI. Notwithstanding, studies so far analyze the P-wave duration from the onset to the offset of the P-waves, thus including both atria indivisibly. By contrast, splitting the P-wave and studying its second half involves focusing on the atrium with the highest impact on the atrial substrate alteration, the LA. This would lead to a more precise noninvasive estimation of the atrial substrate modification after PVI and a deeper understanding of the AF mechanisms, facts that could contribute to the decision-making, leading to the planning of a more efficient follow-up strategy.

The aim of the present study is to demonstrate the relevance of the second P-wave half analysis in investigating the atrial substrate modification provoked by PVI in order to improve the personalized therapy. For this purpose, the alterations of the P-wave temporal characteristics after PVI are assessed, with a special focus on the separate analysis of RA and LA modification, as manifested by the first and second halves of the P-wave, respectively.

## 2. Materials and Methods

### 2.1. Materials

A database of 29 paroxysmal AF patients undergoing circumferential radiofrequency (RF) PVI was employed. Patients had not undergone any PVI procedure in the past and 5-min continuous standard 12-lead ECG recordings at a sampling frequency of 1 kHz were acquired before and after PVI for each patient by a Labsystem™ PRO EP recording system (Boston Scientific, Marlborough, MA, USA). Patients were in sinus rhythm (SR) during the procedure. Recordings before PVI were acquired 7–23 min before the initiation of the PVI procedure (mean time of the initiation of the pre-PVI recording: 12.3±3.6 min before PVI). Recordings after PVI were acquired 1–12 min after PVI (mean time of the initiation of the post-PVI recording: 3.9±5.1 min after confirmation of successful PVI). Isolation was guided by electroanatomical mapping and performed with an ablation catheter encircling the left and right PVs, emitting RF energy to achieve complete electric insulation. The PVI endpoint was AF non-inducibility, confirmed by continuous pacing after the isolation of each PV. In order to facilitate the analysis, lead II was chosen and extracted for processing, since this channel provides P-waves of high amplitude and monophasic positive morphology [37].

### 2.2. Signal Preprocessing

**Figure 1:**
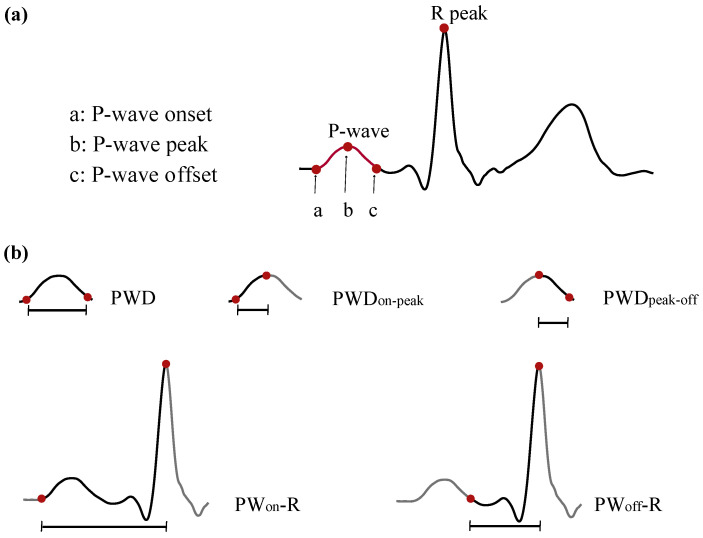
(**a**) Fiducial points of P-waves and R peaks. (**b**) Calculated temporal characteristics.

Preprocessing and analysis were performed with MATLAB© R2019b version. Signal was denoised by a wavelet-based denoising method to remove the powerline interference, followed by a bidirectional low-pass filtering with cut-off frequency at 70 Hz for the muscle noise removal and the removal of baseline wander with a high-pass filter with cut-off frequency at 0.8 Hz [38,39]. Ectopic beats were detected and cancelled in order to be replaced by linearly interpolated beats [40,41]. Although not all recordings contained ectopic beats, in case of presence, they did not exceed the 4% of total beats. Finally, for each recording, P-waves were detected and R-peaks and P-wave fiducial points were defined [42,43], as can be observed in Figure 1a. In normal P-waves, peak (b) was defined as the maximum point of the signal between (a) and (c) of Figure 1a. In case of notched P-waves, the peak was defined as the middle point between the two local peaks.

### 2.3. P-wave Temporal Characteristics

After the detection of fiducial points for each P-wave, the following P-wave temporal characteristics were defined (see Figure 1b):

- P-wave duration (PWD): distance from the P-wave onset to the P-wave offset. Measures the atrial depolarization duration from the beginning of the RA until the end of the LA activation.
- $PWD_{on\text{-}peak}$: distance from the P-wave onset to the P-wave peak. Measures the RA depolarization duration.
- $PWD_{peak\text{-}off}$: distance from the P-wave peak to the P-wave offset. Measures the LA depolarization duration.
- $PW_{on}\text{-}R$: distance from the P-wave onset to the R peak. Measures the atrial depolarization and the atrioventricular conduction durations.
- $PW_{off}\text{-}R$: distance from the P-wave offset to the R peak. Measures the atrioventricular conduction duration independently from the atrial depolarization.

As all of the abovementioned features are affected by heart rate (HR) fluctuations, a correction factor (*CF*) is recommended to compensate for the bias inserted [44]. In this study, the *CF* is based on a 60 bpm HR, which corresponds to an interbeat interval (*IBI*) of 1000 ms (one beat per second). Hence, the *CF* is calculated for the *i*-th P-wave falling within the *i*-th *IBI* as

(1) $$CF_{i}=\frac{1000}{IBI_{i}}.$$

**Figure 2:**
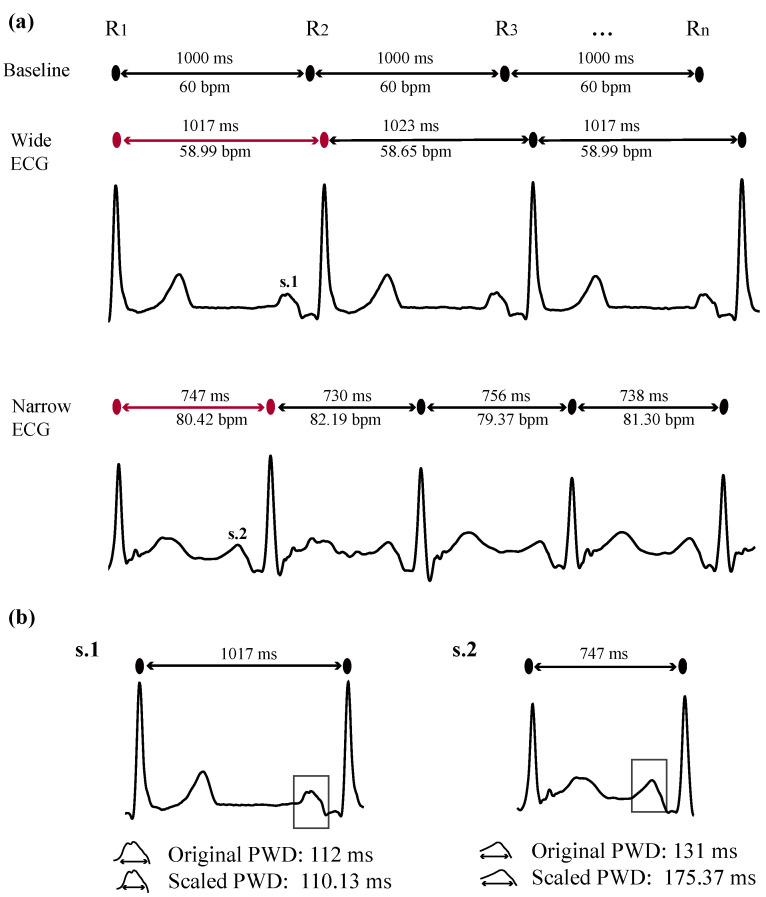
Example of P-wave scaling for interbeat intervals longer or shorter than 1000 ms. (**a**) Baseline interbeat interval at 1000 ms and interbeat intervals of a wide and a narrow ECG. Red intervals show the beats chosen to be analyzed as an example in (**b**). (**b**) PWD scaling for P-waves of a wide (s.1) and a narrow (s.2) signal. Wide signal: P-wave is shrunk after scaling. Narrow signal: P-wave is lengthened after scaling. The remaining features are scaled accordingly.

Figure 2 shows an example of how P-waves belonging to *IBI*s longer or shorter than 1000 ms are scaled according to *CF*. It can be observed that *CF* scales linearly the employed features with respect to HR. Given the direct exposure of atrial tissue to RF energy, it was assumed that atrial components of the ECG are directly affected from the RF applications, as RF energy has a significant effect on HR [45]. Features scaled with the *CF* will be represented in the remaining manuscript as **A**(*x*), where *x* is the name of the various features described previously.

### 2.4. Correlation between PWD and the Remaining Temporal Characteristics

Being PWD one of the most employed features in studies evaluating the atrial substrate alteration, it is used as the baseline of the present study. Correlations between PWD and the remaining features were calculated. For each recording before and after *PVI*, correlations were computed for every single beat with Pearson’s correlation coefficient (PCC) and then averaged across the entire signal. Moreover, correlation of the variation (CoV) caused in features by *PVI* was calculated between PWD and the rest of the characteristics. CoV between PWD and each of the remaining features was calculated by PCC at a recording basis, performing a single calculation for each patient. Given feature *x*, variation due to *PVI* was calculated as follows

(2) $$\Delta \left(x\right)=\frac{x_{after\;PVI}}{x_{before\;PVI}}-1.$$

### 2.5. Statistical Analysis

Data normality and homoscedasticity were tested with Saphiro–Wilk and Levene tests, respectively [46,47]. Results indicated the employment of a Mann–Whitney U-test to compare values before and after PVI. Additionally, median values and variation due to PVI in form of percentage have been calculated by multiplying Equation (2) by 100%.

## 3. Results

**Figure 3:**
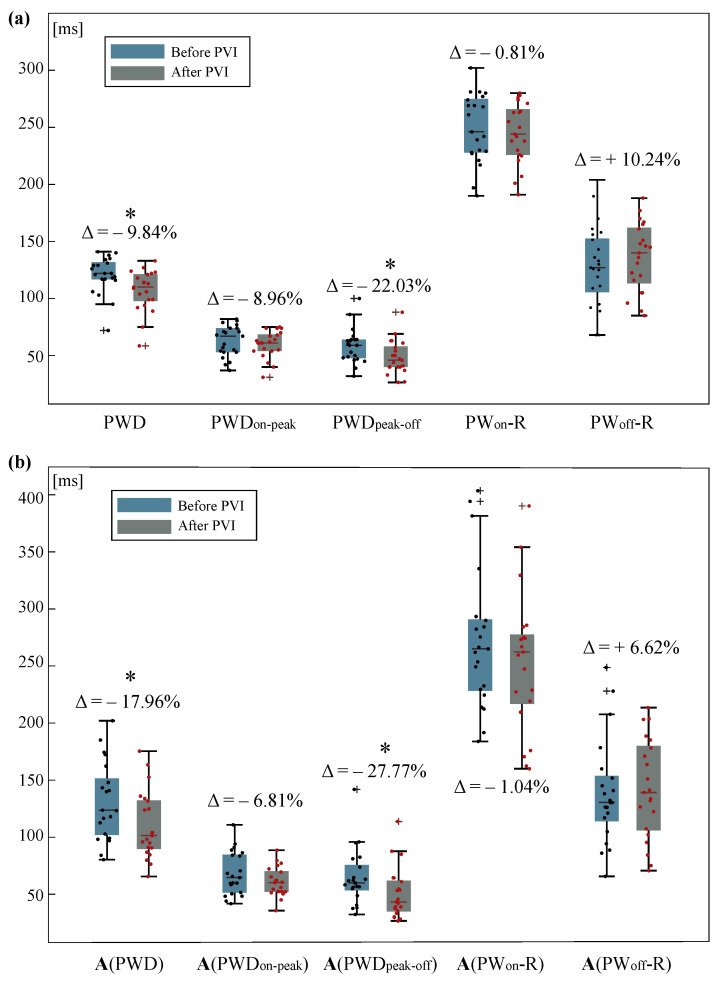
Boxplot with scatterplots for the analyzed features before and after PVI. (**a**) Boxplots for raw features. (**b**) Boxplots for scaled features according to the correction factor. The symbol (•) stands for normal values whereas (+) stands for outliers. Variation due to PVI (Δ) is additionally shown. Significant variations are shown in (*).

Statistical results can be seen in Table 1, where median values and interquartile ranges before and after PVI as well as the variation due to PVI can be observed. These results are further illustrated in Figure 3, where the box and whisker plots of each feature before and after PVI are presented.

PWD decreased significantly due to PVI (Δ=−9.84%, p=0.0085). The same tendency was observed when PWD was scaled by the CF, where the statistical power was weaker, albeit still significant (Δ=−17.96%, p=0.0442). Distance from P-wave peak to P-wave offset was also statistically reduced after the PVI procedure and to a higher degree than PWD (Δ=−22.03%, p=0.0250). Unlike PWD, the statistical power of $PWD_{peak\text{-}off}$ was not affected by scaling while the decreasing tendency remained (Δ=−27.77%, p=0.0268). $PWD_{on\text{-}peak}$ did not shorten significantly regardless of the application of CF (up to −8.96%, p>0.3651). None of the P-R features showed statistically significant variations (p>0.05) either.

**Figure 4:**
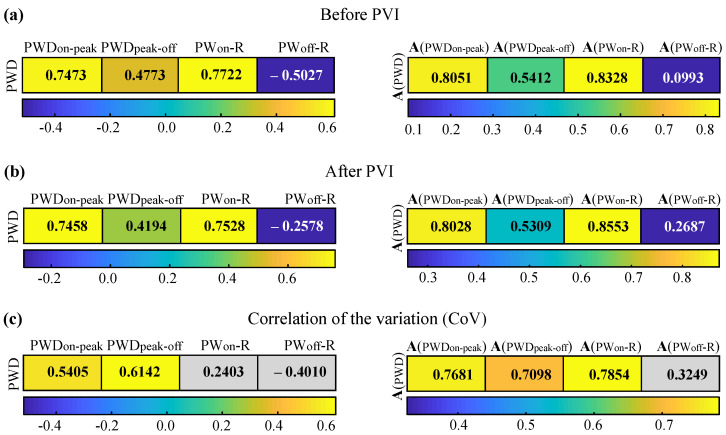
Correlation matrices for the relationship between PWD and the remaining features. Values without scaling are on the left column and with scaling on the right. (**a**) Results before PVI. (**b**) Results after PVI. (**c**) Results for the correlation of the variation. Gray cells show statistically insignificant relationships.

Correlations investigated in recordings before and after PVI showed strong and statistically significant relationships between PWD and the first part of the P-wave ($PWD_{on\text{-}peak}$, before PVI: PCC = 0.747, p<0.0001, after PVI: PCC = 0.746, p<0.0001 ) and between PWD and the interval from the onset of the P-wave to the R-peak ($PW_{on}\text{-}R$, before PVI: PCC =0.772, p<0.0001, after PVI: PCC =0.753, p<0.0001). These relationships were further corroborated after scaling by the CF, as can be observed in Figure 4.

Correlation between PWD and $PWD_{peak\text{-}off}$ was found to be moderate (before PVI: PCC =0.477, p<0.0001, after PVI: PCC =0.419, p<0.0001), with CF slightly potentiating this effect, still to a moderate level (before PVI: PCC =0.541, p<0.0001, after PVI: PCC =0.531, p<0.0001).

A weaker correlation between PWD and all features was found regarding the effect of PVI. For P-wave components, correlation with $PWD_{on\text{-}peak}$ was slightly lower than correlation with $PWD_{peak\text{-}off}$, as can be seen from Figure 4. Nevertheless, application of CF reverted this observation, with $PWD_{on\text{-}peak}$ being again slightly more correlated with PWD than $PWD_{peak\text{-}off}$. While in pre- and post-PVI cases $PW_{on}\text{-}R$ showed notably higher correlation with PWD than the rest of the features, CoV of $PW_{on}\text{-}R$ was remarkably low and insignificant. Notwithstanding, CF application not only boosted this relationship but also led to the highest concordance between the variations observed in $PW_{on}\text{-}R$ and PWD. On the other hand, correlation with $PW_{off}\text{-}R$ was negative before normalization and positive but low after normalization, with both results not being statistically significant.

## 4. Discussion

P-wave duration is a popular tool in studying various cardiac pathologies [48]. In AF, P-wave duration is an evaluator of the conduction delay provoked by fibrotic areas [23,28,29,34]. These areas might be found either in RA or LA or both atria [10]. Nevertheless, RA and LA are not equally affected by PVI, which has a major effect in LA function [21].

Despite the disproportionate effect of PVI on RA and LA, studies insist on evaluating the substrate alterations as a homogeneous phenomenon, complicating the planning of an efficient follow-up treatment for AF individuals. Several works have defined thresholds for prolonged PWD varying from 120 ms to 140 ms, a difference which is not negligent when considering a tissue to be remodelled [23,28,29,34]. Deviations in the cut-off points may stem from different pathologies or AF types present in the database. P-wave delineation may additionally affect the calculated PWD, with a small deviation either in P-wave onset or offset showing significant differences in the results.

Given that LA alteration is the major phenomenon after PVI procedures and the fact that P-wave from lead II can be split in two parts corresponding to the depolarization of each of the atria, a separate analysis of the two P-wave parts would be more meaningful when precision in calculations is an important issue. The present study attempted a separate P-wave first and second part analysis in order to investigate in detail the RA and LA modifications provoked by PVI. Additionally, P-R interval was also decomposed in parts describing separate atrial and atrioventricular conduction. The findings of this novel approach are quite interesting.

Duration of the entire P-wave was significantly shortened after PVI. This observation is in line with a plethora of previous studies [23,24,28,29,31,32]. When analyzing the first and second P-wave parts separately, a significant shortening was only observed in the second part of the P-wave, describing the reduction in LA depolarization time. Not only was the duration of the second P-wave part shortened due to PVI, but also the statistical power of this alteration remained unaffected from scaling by the CF. By contrast, statistical power of the entire P-wave was moderated after scaling. Among many formulas offered in the literature, a linear model was chosen for the normalization, as linear scaling shows less correlation with HR, hence being less HR-dependent [49,50,51]. Nevertheless, the choice of other formulas can be adopted without altering the main findings of the analysis. At the same time, the first part of the P-wave remained almost intact regardless of the CF, implying a much lower impact of PVI on RA function. It should also be highlighted that the degree of shortening of the second part of the P-waves was the highest among all employed characteristics. This stresses the importance on focusing on the second P-wave part, which is in fact the principal source of the P-wave shortening that is reported by many studies.

The significance of the second P-wave part in clinical environment was recently appreciated in a study that investigated the correlation of P-wave parameters with inter- and intra-atrial conduction times measured via invasive analysis, finding a relatively high correlation between the second P-wave part and LA conduction times [52]. The study additionally used surface and invasive parameters to discern between AF occurrence and non-AF occurrence during electrophysiological studies of a randomly chosen set of patients. The authors found the second part of the P-wave to be a good independent predictor for AF occurrence. These results corroborate the findings of the present study, implying a direct relationship between the second P-wave part and pathophysiologic phenomena observed during AF, thus highlighting the importance of studying the P-wave separately.

Despite the fact that the second but not the first part of the P-waves got significantly shortened after PVI, it was the first part that correlated in a stronger way with the entire P-wave duration. Normal sinus rhythm heartbeat starts in sinus node, which is the first site to be depolarized and then is propagated through other right atrial sites towards the LA [35,36]. Sinus node plays a significant role in P-wave morphology. Additionally, it takes more time for the right than the left atrial depolarization, as described in Section 3. These facts may lead to a higher overall correlation between the duration of the first P-wave part and the duration of the entire P-wave. Therefore, when the alteration of the entire P-wave duration is assessed, RA depolarization may affect the result, masking the degree of modification of the LA function, which should be the primal focus point. This observation explains the lower shortening rate of the entire P-wave duration with respect to the shortening of the second half of the P-wave and the fluctuations in statistical power when CF has been applied for PWD.

P-wave duration showed the highest correlation with the P-R interval, when measured from the initiation of the RA depolarization until the R-peak. At the same time, P-R part measured from the end of the LA depolarization until the R-peak not only showed the lowest correlations but also marked insignificant results, when the PVI effect was assessed. This implies the fact that P-R interval is highly dependent on the atrial activity and does not exclusively express the atrioventricular conduction. In fact, although $PW_{on}\text{-}R$ has shown a negligible and nonsignificant reduction after PVI, $PW_{off}\text{-}R$ seems to have been increased. Therefore, P-R prolongation reported in previous studies may actually be caused by prolongation of the atrial depolarization time and its interpretation may be misleading. These results are in line with previous studies dealing with this issue, where additionally a correlation between short $PW_{off}\text{-}R$ and AF incidence has been found in long $PW_{on}\text{-}R$, as prolongation is provoked by PWD lengthening in this case [12,25]. These findings highlight the necessity of partial analysis of the atrial and atrioventricular ECG components rather than studying the P-wave and P-R interval indivisibly.

The hypothesis that the P-R interval, thought to assess the atrioventricular conduction, is highly dependent on the atrial depolarization has led a previous work to the study of the separate atrial and atrioventricular components of the P-R interval, which coincide with the features of the present study [12]. Interestingly enough, a prolongation of the first P-wave part, corresponding to the RA depolarization, has been found to be associated with AF in a stronger way than the prolongation of the LA P-wave component. It should be highlighted, however, that substantial differences exist between the aforementioned and the present study.

In the first place, the utilized database also included patients with pathologies other than AF, while AF patients would mostly fall into the persistent AF category. Different pathologies or different AF types may also be manifested by RA conduction slowing. As mentioned afore, P-wave duration prolongation is not an exclusive paroxysmal AF phenomenon [48] and hence, connection between prolongation of the first half of the P-wave and AF incidents can be observed. Additionally, recordings were not obtained during PVI procedure. The main scope of the current study is a high-definition analysis on how PVI alters the atrial substrate, by looking deeper into the exact effect on each atrium separately. Consequently, the main finding of the present study is the shortening and significant alteration of LA and the significance of the study of $PWD_{peak\text{-}off}$ in atrial substrate modification evaluation after PVI. Finally, while P-wave duration and duration of the first P-wave part were calculated in the same way as in the present study, a different, indirect strategy was recruited to compute the second part of the P-wave. Since a straightforward calculation of the second part of the P-wave is missed, a deviation between the real and estimated LA depolarization time may exist.

In radiofrequency sessions, heart rate fluctuations are observed as an outcome of the autonomous nervous system stimulation [45]. These alterations may in turn affect the temporal P-wave and P-R features [44]. In a previous study, P-R prolongation was connected with lower heart rate [25]. Therefore, when dealing with temporal features, a scaling is suggested in order to unmask any potential changes that are distorted by the variable heart rate. This aspect was taken into consideration in the current analysis, by adding an extra step of scaling each feature with respect to a 60 bpm heart rate. Although it did not affect significantly the statistical results of the pre-post analysis, it did play a significant role in the assessment of the correlations between PWD and the remaining features, potentiating each and every relationship, especially when the PVI-induced variation was examined. This way it was possible to identify the high correlation between the PVI effect on P-wave duration and on P-R interval, which would otherwise be ignored, thus showing a misleading lack of connection between the P-R interval and the atrial depolarization modification due to PVI.

The aforementioned aspects highlight the importance of reconsidering the way that the atrial substrate alterations after PVI are being evaluated in paroxysmal AF patients from surface ECG recordings. The present study has provided an in-detail perspective of how atrial depolarization time is shortened after PVI, with LA conduction time being the center of this modification. It is demonstrated that studying the second half of the P-wave can lead to a more accurate evaluation of the atrial substrate alteration, one of the most controversial and delicate issues in planning personalized AF follow-up strategies. Moreover, studying exclusively the second P-wave part facilitates the delineation process. As one of the two fiducial points that need to be specified, the P-wave peak, can be reliably and easily detected, the ambiguity regarding the partial P-wave delineation is significantly lower with respect to the entire P-wave delineation. Therefore, adopting the aforementioned strategy can facilitate the procedure and enhance the precision and robustness of the substrate alteration estimation.

## 5. Conclusions

The second part of P-waves is the most relevant in evaluating the atrial substrate modification from surface recordings after PVI, outperforming the entire P-wave analysis. Hence, splitting the P-wave in two parts and focusing on the second P-wave part is highly recommended. For the assessment of the atrioventricular conduction alterations, the atrial component should be subtracted. Scaling is a necessary step for studies investigating the correlation between depolarization time of various atrial and atrioventricular components and should also be considered by future studies.

## Figures and Tables

**Table 1:** Statistical analysis for P-wave features before and after PVI. Median values, interquartile range (IQR) and variation due to PVI. Features with statistically significant differences due to PVI are shown in **bold**.

		Median Values (IQR)		
**Feature**	p	**Before PVI**	**After PVI**	Δ **[%]**
PWD	0.0085	122.00(12.00)	110.00(11.00)	−9.84
$PWD_{on\text{-}peak}$	0.5289	67.00(14.00)	61.00(11.00)	−8.96
${\mathrm{PWD}}_{\mathrm{peak}\text{-}\mathrm{off}}$	0.0250	59.00(8.00)	46.00(7.00)	−22.03
$PW_{on}\text{-}R$	0.5585	246.00(10.00)	244.00(11.00)	−0.81
$PW_{off}\text{-}R$	0.3519	127.00(8.00)	140.00(7.00)	+10.24
A(PWD)	0.0442	123.63(15.08)	101.42(12.15)	−17.96
**A**($PWD_{on\text{-}peak}$)	0.3651	64.59(13.00)	60.19(10.97)	−6.81
$A({\mathrm{PW}}_{\mathrm{peak}\text{-}\mathrm{off}})$	0.0268	59.89(8.55)	43.26(7.55)	−27.77
**A**($PW_{on}\text{-}R$)	0.3924	264.96(14.11)	262.19(12.73)	−1.04
**A**($PW_{off}\text{-}R$)	0.6507	130.47(9.00)	139.11(9.62)	+6.62

## Data Availability

The data supporting reported results and presented in this study are available on request from the corresponding author.
